# Dendritic Cell‐T Cell Crosstalk Mediated Cancer Immunotherapy

**DOI:** 10.1002/exp2.70134

**Published:** 2026-02-16

**Authors:** Ying Cai, Jiameng Chen, Yaping Li

**Affiliations:** ^1^ State Key Laboratory of Drug Research and Center of Pharmaceutics, Shanghai Institute of Materia Medica Chinese Academy of Sciences Shanghai China; ^2^ University of Chinese Academy of Sciences Beijing China

1

Adequate T cell infiltration and activity maintenance are critical in antitumor immunity. T cell exhaustion largely leads to its dysfunction in tumors and is difficult to reverse. Therefore, most previous studies have tended to regulate T cell function through immune checkpoint blockade or chimeric antigen receptor T cell therapy [1]. However, these therapies are affected by tumor heterogeneity and immunosuppression, which remain challenging to achieve broad and consistent therapeutic efficacy across diverse patient populations and may lead to drug resistance and toxicity [2, 3].

Dendritic cells (DCs) in tumor‐draining lymph nodes (DLNs) present antigens to naïve T cells, thereby overcoming immunological tolerance. In the tumor microenvironment (TME), DCs sustain T cell survival and drive their effector differentiation, consequently amplifying local antitumor immunity [4]. Accordingly, DC vaccines and in situ vaccination approaches have been explored in cancer therapy, especially for hematological malignancies. However, their clinical utility remains limited by weak cellular immune responses and high production costs [5].

There is increasing evidence that supports that DC‐T cell interactions are critical in the cancer‐immunity cycle, which mediate a series of key reactions in TME and DLNs [6]. Mutualism between DCs and T cells, based on reciprocal signals exchanged during physical interactions, is essential for tumor control and successful cancer immunotherapy [4]. It has been demonstrated that T cell activation induced by programmed cell death protein 1 (PD‐1) immune checkpoint blockade monoclonal antibody treatment depends on the crosstalk between DCs and T cells. In this process, DCs sense interferon‐γ released from neighboring T cells and produce interleukin‐12 to potentiate antitumor T cell immunity [7]. Despite the central role of DC‐T crosstalk in initiating antitumor immunity, the limited efficacy of current DC vaccines, the persistent challenge of T‐cell exhaustion, and the lack of therapeutics directly targeting the DC‐T interface have left this important immunological axis insufficiently explored.

Reporting in *Cell*, Rony Dahan and colleagues now demonstrate a bispecific DC‐T cell engager (BiCE) designed to strengthen physical interactions between conventional type 1 dendritic cells (cDC1s) and PD‐1^+^ T cells [8]. This design is based on the observation that DC frequency in human tumors is directly associated with the positive responses to PD‐1 blockade therapy, particularly the abundance of cDC1s. The formation of DC‐T cell doublets in both tumor and DLN sites was enhanced following BiCE injection, leading to the increased maturation of cDC1s, activation and proliferation of CD8^+^ T cells and further reprogramming of the tumor‐infiltrating lymphocyte composition (Figure 1). BiCE consistently induced a strong antitumor response and exhibited therapeutic efficacy across various aggressive tumor models, whether administered alone or in a combined therapy. Notably, its activity extended to both primary and metastatic tumors, including those unresponsive to traditional aPD‐1 blockade treatment. One important implication of this work is that rather than relying on complex cellular therapies or cytokine‐ and chemokine‐based modulation of the immune microenvironment, a simple and rational design that reduces the physical distance between DCs and T cells can effectively harness inherent immune cells to amplify antitumor immunity. Although this work verifies that directly mediating the physical connection between DCs and T cells can enhance antitumor immunity, there are some limitations that should be considered. In the design of BiCE, differences in the affinity and specificity of the Fab arms may lead to variations in the types of cells being bridged, the extent of contact, and the duration of the interaction, thereby introducing non‐negligible challenges for the production and manufacturing of bispecific antibodies. Apart from C‐type lectin domain family 9 member A (CLEC9A)^+^ cDC1s and PD‐1^+^ T cells, how other cells expressing related ligands contribute to this process, especially those engaged in antitumor immunity, remains to be determined. Potential risks associated with BiCE treatment, ranging from acute excessive inflammation to long‐term complications such as immune tolerance, therapeutic resistance, and tumor‐intrinsic immune‐evasion mechanisms, should be further investigated. As this strategy has so far been demonstrated only in murine models, evaluating its potential for translation into human cancer therapy will be a crucial next step, including the de novo design of bispecific antibodies optimized for human immune cell phenotypes. Moreover, interpatient variability in cDC1 abundance may further hinder the clinical translation of this approach.

**FIGURE 1:**
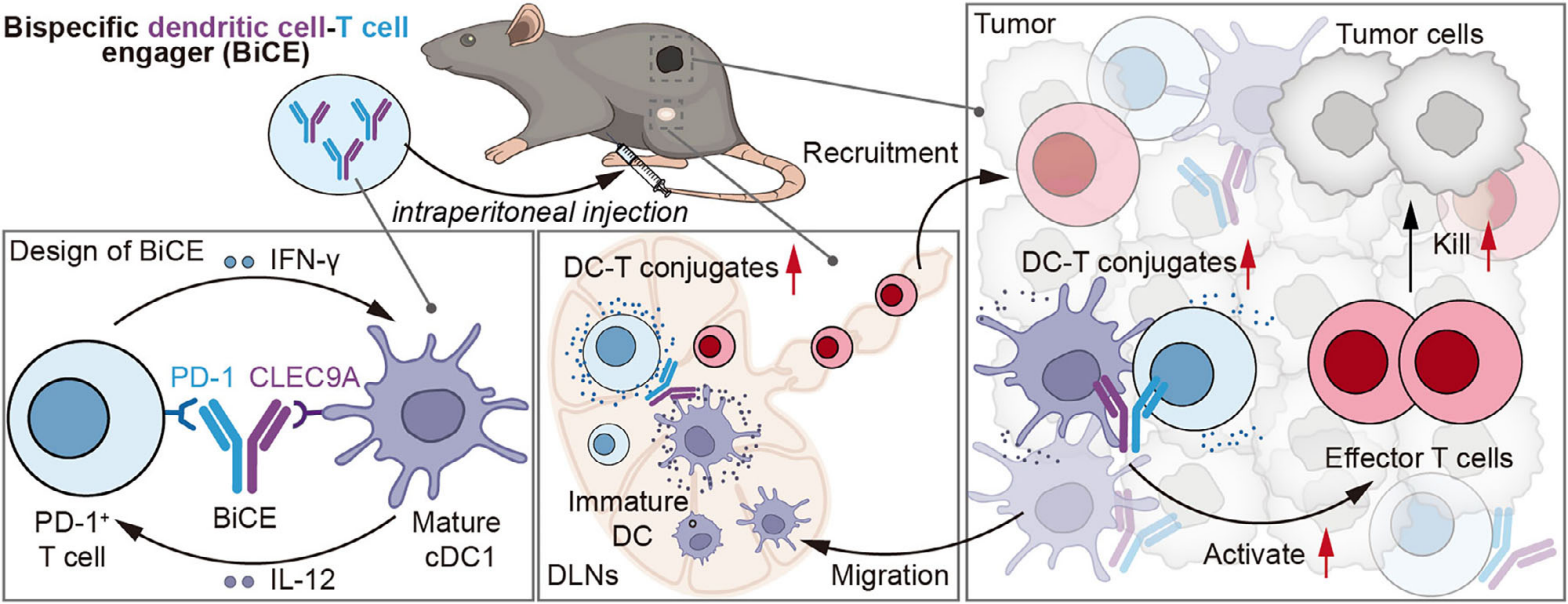
Schematic illustration of the design and mechanism of bispecific DC‐T cell engager. BiCE exhibits dual specificity for PD‐1 expressed on T cells and CLEC9A expressed on cDC1s. It effectively mediates the formation of DC‐T cell conjugates within both the tumor and tumor‐draining lymph nodes, thereby promoting T cell activation and effector T cell differentiation. Migratory cDC1s at the tumor site can traffic to the draining lymph nodes, further amplifying antitumor immune signaling and contributing to the elimination of tumor cells. BiCE, bispecific dendritic cell‐T cell engager; PD‐1, programmed cell death protein 1; CLEC9A, C‐type lectin domain family 9 member A; DC, dendritic cell; cDC1, conventional type 1 dendritic cell; IFN‐γ, interferon‐γ; IL‐12, interleukin‐12; DLNs, draining lymph nodes.

BiCE is a special kind of immune cell engager that induces the crosstalk between DCs and T cells, distinguishing from conventional CD3‐based T cell engager that triggers direct cytotoxic activation [9]. By strengthening endogenous DC functionality in a manner consistent with natural immunobiology, BiCE promotes more effective antitumor immunity without requiring homogeneous tumor‐antigen expression and reduces the likelihood of systemic T‐cell hyperactivation.

## 2 Perspective

**FIGURE 2:**
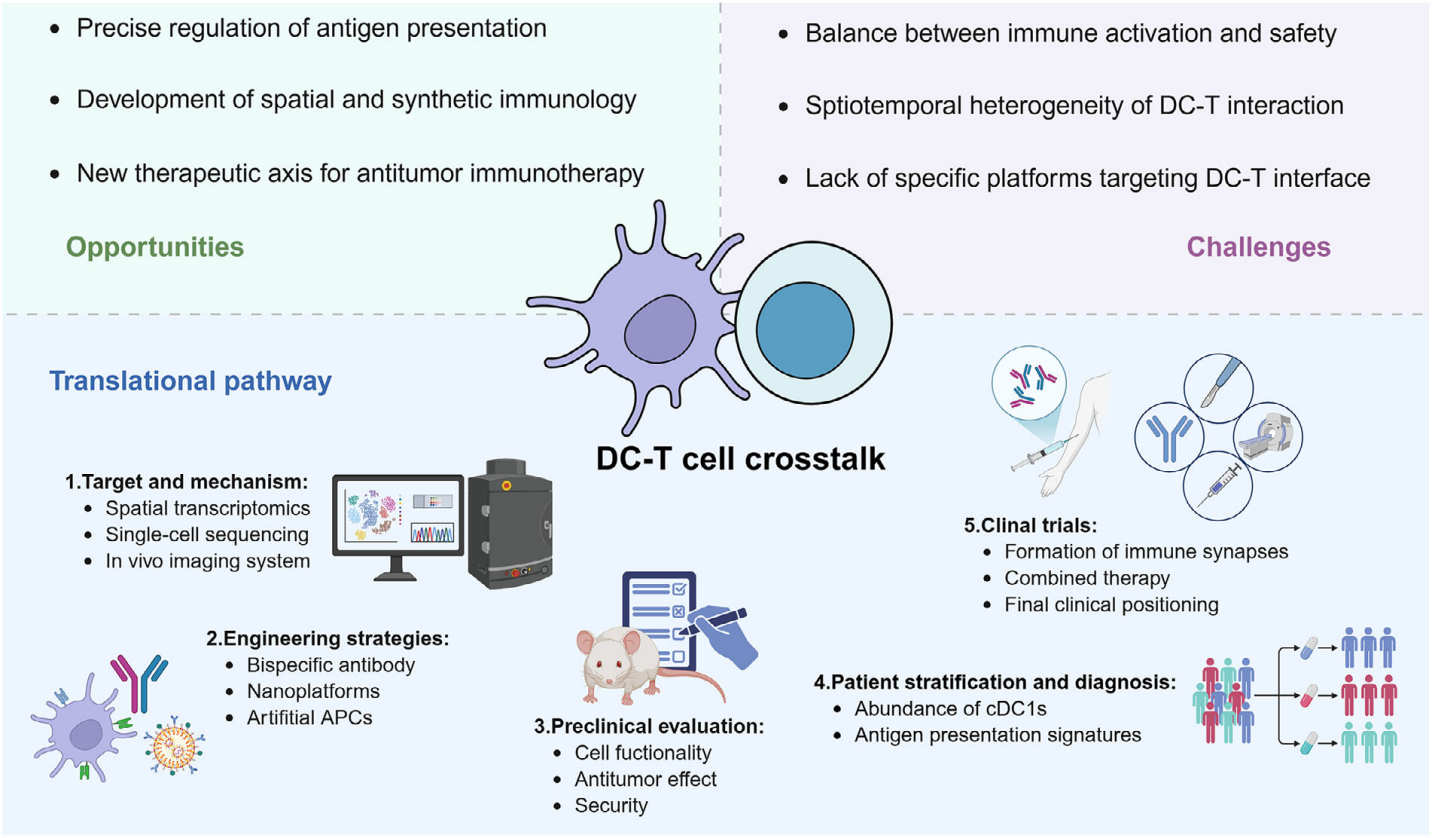
Overview of the key opportunities, challenges, and translational pathways in targeting DC‐T cell crosstalk for cancer immunotherapy. Key opportunities include precise control of antigen presentation and advances in spatial and synthetic immunology, while major challenges arise from safety concerns, interaction heterogeneity, and the lack of specific targeting platforms. Advances in emerging technologies will be essential for its translational pathway spanning target discovery, engineering strategies, preclinical evaluation, patient stratification, and clinical trial development. Created with BioRender.com and reprinted with permission.

In the context of next‐generation immunotherapies, BiCE occupies a distinctive position as a programmable tool for precisely modulating immune cell interactions, which is expected to provide a new therapeutic axis for cancer immunotherapy. The crosstalk between DCs and T cells represents a highly complex and dynamic process. Distinct subsets within both populations play specialized roles across different stages of antitumor immunity [10]. Moreover, during priming, individual T cells likely engage with multiple DCs in a temporally and spatially coordinated manner, adding another layer of complexity to this interaction [11]. As spatial immunology continues to reveal the microanatomical organization of immune responses [12], BiCE offers a means to control key cell–cell contacts with temporal and spatial precision. Moreover, its modular architecture aligns with synthetic immunology, allowing integration with artificial receptors or molecular switches for logic‐controlled immune responses [13]. Supported by emerging technologies, future research should prioritize the clinical translation of strategies that modulate DC‐T cell crosstalk, encompassing the identification of new targets and mechanisms, the development of engineering approaches, rigorous preclinical evaluation, patient stratification and diagnostic frameworks, and the design of clinical trials and combination therapies (Figure 2). Remarkably, BiCE simplifies immunotherapeutic approaches, aligns with the body's intrinsic immune processes, and provides a compelling proof of principle for the application of bispecific antibodies in cancer immunotherapy. Translating this concept into human studies could provide valuable insights into its therapeutic potential.

## Author Contributions

Ying Cai and Jiameng Chen wrote the original manuscript. Ying Cai and Yaping Li revised the manuscript.

## Conflicts of Interest

The authors declare no conflicts of interest. Yaping Li is a member of the *Exploration* editorial board, and he was not involved in the handling or peer review process of this manuscript.

## Data Availability

Reprints and permissions information are available.
